# Analysis of Fine Motor Skills in Essential Tremor: Combining Neuroimaging and Handwriting Biomarkers for Early Management

**DOI:** 10.3389/fnhum.2021.648573

**Published:** 2021-06-08

**Authors:** Karmele Lopez-de-Ipina, Jordi Solé-Casals, José Ignacio Sánchez-Méndez, Rafael Romero-Garcia, Elsa Fernandez, Catalina Requejo, Anujan Poologaindran, Marcos Faúndez-Zanuy, José Félix Martí-Massó, Alberto Bergareche, John Suckling

**Affiliations:** ^1^Department of Psychiatry, University of Cambridge, Cambridge, United Kingdom; ^2^EleKin Research Group, Department of System Engineering and Automation, University of the Basque Country UPV/EHU, Donostia-San Sebastian, Spain; ^3^Data and Signal Processing Research Group, University of Vic-Central University of Catalonia, Barcelona, Spain; ^4^Cajal Institute, Consejo Superior de Investigaciones Científicas (CSIC), Madrid, Spain; ^5^The Alan Turing Institute, British Library, London, United Kingdom; ^6^Tecnocampus, Universitat Pompeu Fabra, Mataro (Barcelona), Spain; ^7^Neurodegenerative Disorders Area, Biodonostia Health Research Institute, Donostia-San Sebastian, Spain; ^8^Movement Disorders Unit, Department of Neurology, Donostia University Hospital, Donostia-San Sebastian, Spain; ^9^Biomedical Research Networking Centre Consortium for the Area of Neurodegenerative Diseases (CIBERNED), Madrid, Spain

**Keywords:** essential tremor, fine motor skills, neuroimaging, handwriting, early management

## Abstract

Essential tremor (ET) is a highly prevalent neurological disorder characterized by action-induced tremors involving the hand, voice, head, and/or face. Importantly, hand tremor is present in nearly all forms of ET, resulting in impaired fine motor skills and diminished quality of life. To advance early diagnostic approaches for ET, automated handwriting tasks and magnetic resonance imaging (MRI) offer an opportunity to develop early essential clinical biomarkers. In this study, we present a novel approach for the early clinical diagnosis and monitoring of ET based on integrating handwriting and neuroimaging analysis. We demonstrate how the analysis of fine motor skills, as measured by an automated Archimedes’ spiral task, is correlated with neuroimaging biomarkers for ET. Together, we present a novel modeling approach that can serve as a complementary and promising support tool for the clinical diagnosis of ET and a large range of tremors.

## Introduction

Essential tremor (ET) is a highly prevalent movement disorder that greatly impacts an individual’s quality of life. ET affects both males and females equally (Louis, 2010; Louis and Ferreira, 2010; Benito-Leon, 2014). While ET can affect the voice, head, and lower extremities (Avecillas-Chasin et al., 2018), hand tremor is the predominant concerning symptom as it occurs in nearly all cases. Specifically, hand tremor produces a deterioration of fine motor skills due to a presumed cerebellar neurodegenerative process (Brittain and Brown, 2013; Jellinger, 2014; Louis, 2018; Louis et al., 2020; Sepúlveda and Fasano, 2020).

Recently, an international task force defined tremors as an involuntary, rhythmic, oscillatory movement of a body part (Bhatia et al., 2018). It is important to note that the limbs and head, when unsupported, exhibit minimal tremor, referred to as physiological tremor. Physiological tremor is generally not visible or symptomatic unless it is enhanced by fatigue or anxiety, whereas pathological tremor is usually visible and persistent. Several movement disorders need to be differentiated from tremor. ET has a prevalence of 4.6% among people aged 65 and older (Louis and Ferreira, 2010) and is defined as requiring at least a 3-year history of tremor and excludes isolated head and isolated voice tremors. Moreover, a 3-year history is included to reduce the odds of subsequent development of other neurological signs (e.g., dystonia, parkinsonism, or ataxia). Even with this safeguard, patients with ET may ultimately develop other symptoms, subsequently defined as a combined tremor syndrome, not ET, which represents a major diagnostic challenge (Amlang et al., 2020).

The Archimedes’ spiral is the “the gold standard” reference test for the clinical diagnosis of ET (Pullman, 1998). Initially, these analyses were carried out offline, without the use of any technological tools. Today, technological devices such as digitized tablets and pens are low-cost tools that can be used in clinical practice to complement the diagnosis and monitoring of ET. The first use of such technologies appeared in the 1990s (Elble et al., 1996; Riviere et al., 1997; Pullman, 1998), with rapid developments in more recent times (Miralles et al., 2006; Zeuner et al., 2007; Haubenberger et al., 2011; Louis et al., 2012). These new devices analyze not only classical features, such as the cartesian coordinates (*x*, *y*) of features but also others such as pressure and grip, providing a comprehensive set of biomarkers from in-air and on-surface trajectories (Faundez-Zanuy, 2007; Sesa-Nogueras et al., 2012; Likforman-Sulem et al., 2017). As part of a larger cross-sectional study for characterizing ET, our group has previously demonstrated how nonlinear biomarkers from handwriting (Lopez-de-Ipina et al., 2016, 2018) can be used as a tool for developing an automatic classification of ET toward early diagnosis. Additionally, Solé-Casals et al. (2019) demonstrated how new biomarkers from drawings and handwriting can be utilized from discrete cosine transformations (DCT).

Motor skills can be divided into two groups: gross motor skills, which include larger movements of the arms, legs, feet, or the entire body (crawling, running, and jumping), and fine motor skills (FMS), which are smaller actions, such as grasping an object between the thumb and a finger or using the lips and tongue to taste. In the drawing precision of Archimedes’ spiral, FMS areas are critically involved and regulated by the cerebellum (Miall et al., 2001). Both types of motor skills usually develop together because many activities depend on the coordination of gross and fine motor skills. However, studies have generally shown that a broader definition of FMS, focusing on skill at performing a range of FMS requiring manual dexterity (e.g., pegboard and bead-threading tasks), relates to cognition above and beyond pure speed-driven tasks (e.g., key tapping) or broader hand–eye coordination tests (Brookman et al., 2013; Martzog, 2015). In addition, FMS includes graphomotor skills (GS) including the control and strength of the muscles (Levine, 1987) requiring hand–eye coordination, transformation of a visually perceived object into motor output, skills involved in writing, and even handwriting (Bart et al., 2007).

**Figure 1:**
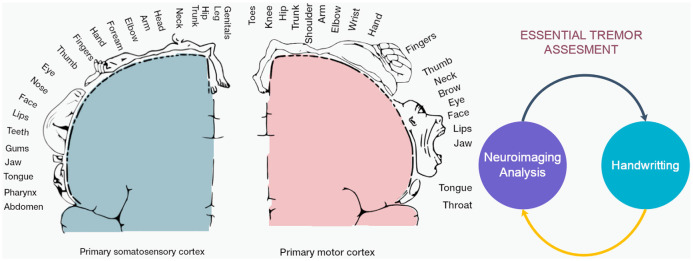
This study analyzed the correlation between the indirect signal from a digital tablet and the structural changes in cortical and subcortical areas in the brain. (Left) Cortical homunculus with details of primary somatomotor and motor cortex involved in ET and handwriting. (Right) Diagram of integration of neuroimaging and handwriting analysis for clinical assessment.

In the case of ET, the impairment of fine motor control can be assessed in handwriting and drawing tasks due to pathological overactivity in the cerebello-thalamo-cortical loop (Klaming and Annese, 2014). This overactivity could appear in motor control trajectory, pressure, and/or their combinations. Thus, tracking these subtle differences in phenotype could be useful for early detection and correlation with brain structural changes (Figure 1; Lopez-de-Ipina et al., 2018; Solé-Casals et al., 2019).

**Figure 2:**
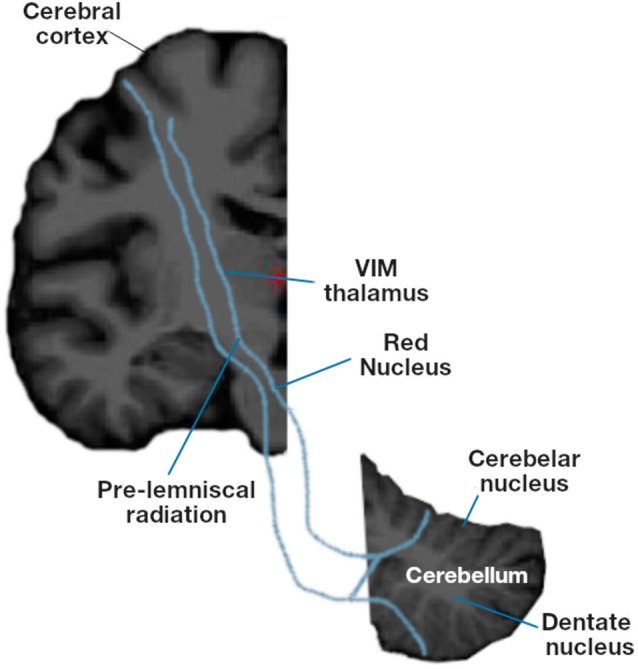
Motor circuit involved in essential tremor: subcortical areas (cerebellum, thalamus and basal ganglia), white matter (cerebellar and cerebellum), motor and premotor cortices.

This study presents a novel approach to diagnosing ET based on the automatic analysis of the Archimedes’ spiral task and structural neuroimaging of the motor circuit and functional networks in the cerebellum (Figures 1, 2). We analyzed the correlation between the indirect signal from a digital tablet and the structural changes in subcortical areas involved in the putative tremor–genesis circuit (cerebellum, thalamus, and basal ganglia), white matter (cerebellar and cerebellum), motor and premotor cortices, and functional networks (Figure 1). This article is organized as follows: the first section contains the *Introduction*, the second section describes the *Materials and Methods*, the third section presents the *Results*, and in the fourth section, the *Discussion* is developed. Finally, the *Conclusions* is presented in the last section.

## Materials and Methods

### Participants

Fifty patients with ET (EtG) and 25 healthy controls (CoG) were recruited from a descriptive study of familial and sporadic ET cases conducted at the Movement Disorders Unit at the Donostia University Hospital, San Sebastian, Spain, from January 2015 to June 2017. All participants were diagnosed by a movement disorder specialist based on the established clinical criteria (Fahn et al., 1993; Hallett, 1998; Bergareche et al., 2015). Historical data, including age, age at onset (AAO), gender, handedness, disease duration, and clinical symptoms, were collected using standard questionnaires.

All participants in this study underwent a series of structured questionnaires and a comprehensive neurological and neuropsychological assessment conducted by three experienced movement disorder specialists. Each patient received a diagnosis of ET after the first evaluation from a neurologist specializing in movement disorders. This diagnosis was subsequently confirmed by consensus with the clinical team based on a review of the available data and electrophysiological records from the second evaluation using the established diagnostic criteria. The Fahn–Tolosa–Marin rating scale score, the Tremor Rating Scale (TRS; Fahn et al., 1993; Tuite and Dagher, 2013), was used to assess patients with ET. The TRS is a widely accepted general scale used in clinical trials. This scale contains three sections: A, to assess the amplitude of the resting, postural, and kinetic tremor at specific anatomical locations; B, for the writing, drawing, and pouring tremor; and C, for activities of daily living. It also has a global assessment by the patient and the examiner, and each item is rated on a scale of 0 to 4. Healthy controls were excluded if they had any neurological illness or family history of ET after clinical evaluation and medical records review. After being given a complete description of the study, all participants provided written and verbal informed consent prior to any procedures. Demographic and clinical data from EtG are summarized in Table 1. No significant differences were found in age, gender, or cognitive performance measured with the Montreal Cognitive Assessment (MoCA) among the CoG and EtG groups in the whole dataset. The study was approved by the Ethics Committee of the Donostia University Hospital.

**Table 1:** Demographic data for handwriting and neuroimaging.

	CoG (*N* = 25)	EtG (*N* = 50)	EtG-HW (*N* = 19)
Age	60.04 ± 13.73	61.16 ± 13.52	62.12 ± 15.68
Gender (M:F)	14:11	25:25	9:8
Tremor level	0 ± 0	13.68 ± 6.45	17 ± 14.18

In this work, the sample consisted of 19 individuals with ET (EtG-HW, *N* = 19, *age* = 62.12 ± 15.68, *range* = 36–81 years). All of the subjects were selected from the full dataset (EtG) and were able to perform the handwriting trial. In order to obtain a balanced dataset, a wide range of tremor severity in patients was selected. The other subjects excluded could not complete the task due to the intensity of the tremor. As larger brains are more likely to exhibit increased gray matter volumes (Zhang and Sejnowski, 2000), associations between regional volumes and tremor level were established using Pearson’s partial correlation (PC) after control for total brain volume effects. This modeling strategy has been extensively used for determining meaningful associations between regional metrics and behavioral phenotypes (O’Brien et al., 2011). There was no significant relationship between tremor level and age across ET patients (*PC* = 0.357, *p*-value = 0.134) between level and age. In this work, brain volume was used as a control variable in PC.

### Imaging Acquisition and Processing

All participants were scanned on a 3-T MRI scanner (MAGNETOM Trio Tim, Siemens Medical Systems, Germany) at the Donostia University Hospital and CITA Alzheimer Foundation in Donostia, Spain. This system used an image matrix coil (TIM) with 32 RF channels providing high-quality image with integrated parallel acquisition techniques. High-resolution T1-weighted images were acquired with the MPRAGE 3D protocol (repetition time, TR = 2,300 ms; echo time, TE = 30 ms; inversion time, TI = 900 ms; field-of-view, FOV = 244 × 244 mm^2^; 1 mm iso-tropic voxels). For the Archimedes’ spiral test, 19 patients with a long range of tremor were selected.

**Figure 3:**
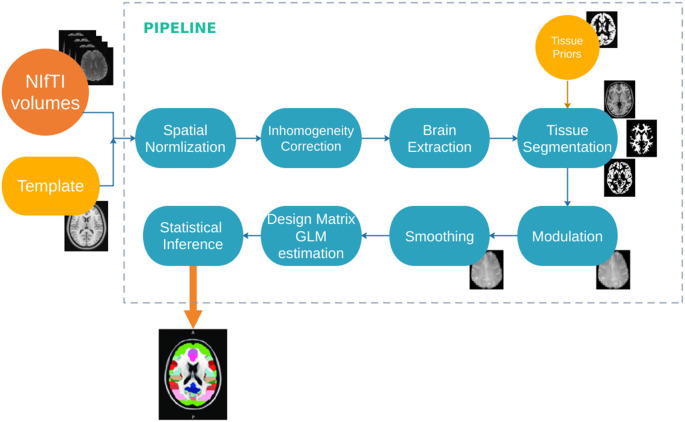
Diagram of the image processing pipeline.

An overview of the image preprocessing pipeline is given in Figure 3. Images were processed by FreeSurfer to estimate regional cortical thickness (CT) and cortical volume (CV) from a three-dimensional cortical surface model derived using intensity and continuity information for cortical and subcortical areas (Fischl and Dale, 2000; Smith et al., 2004; Sharifi et al., 2014; Rosen et al., 2018).

Next, each individual’s cortex was parcellated in the regions defined in the Desikan–Killiany atlas (DSK; Desikan et al., 2006). The result was 308 regions of approximately equal size (500 mm^2^ each) were obtained. This parcellation atlas was constructed with the standard FreeSurfer template (fsaverage) by a backtracking algorithm that subdivided the regions in DSK so that the final parcels were constrained by the original anatomical boundaries (Romero-Garcia et al., 2012; FreeSurfer, 2020). The parcellation was spatially mapped from the standard stereotactic coordinate system of the Montreal Neurological Institute (MNI) to each individual’s MPRAGE acquisition space using surface-based markers. This approach provides better alignment of cortical landmarks than volume-based registration (Andersson and Smith, 2007; Douaud et al., 2007). Moreover, registering patient’s brains to a common space does not result in an age-associated bias making it feasible to accurately compare structural properties and patterns (Ghosh et al., 2010). Finally, the cerebellum was parcellated by the Yeo 7 and 17 functional networks (Buckner et al., 2011; Yeo et al., 2011).

### Handwriting Processing

**Figure 4:**
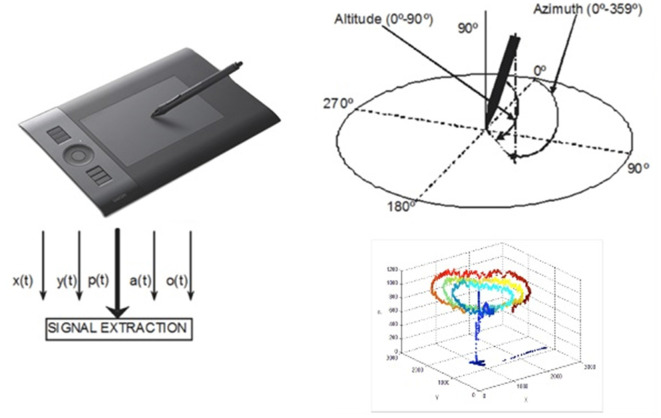
Diagram of the handwriting acquisitions system and example of recorded signals.

The data acquisition system was hosted on a digitizing tablet: the Intuos WACOM 4 2017 (Figure 4), which captured the spatial coordinates (on the surface and in the air), the azimuth and altitude angles of the pen on the tablet, and the pressure is exerted on the surface. The sampling frequency was set to 100 Hz. From the handwriting data, we could extract other variables such as acceleration and speed (Jain et al., 1999; Sadikov et al., 2014). The analysis was done in Matlab using in-house custom software (MATLAB, 2020).

### Statistical Analysis

**Figure 5:**
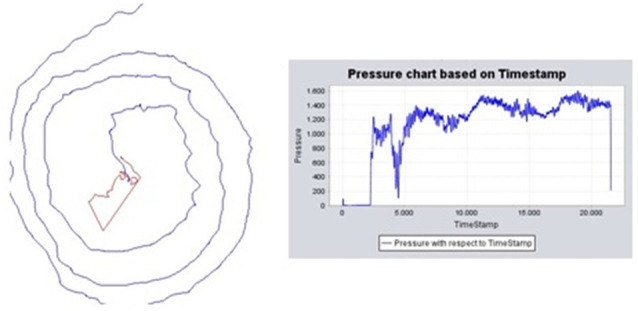
Handwriting processing. (Left) Example of Archimedes’ spiral of an ET patient, trajectory on the surface (blue) and in-air (red), and pressure chart based on the time-stamp. (Right) Pressure chart across time.

The statistical design of the study involved Pearson’s PC using SPSS (SPSS Statistics, 2020) software. A partial correlation is the correlation between an independent variable and a dependent variable after the linear effects of other variables have been removed from both the independent variable and the dependent variable (SPSS-PC). In this work, brain volume was used as the control variable. Then, two experiments were carried out:

1. *Level and handwriting features*. The spatial coordinates (*x*, *y*), the pressure, the azimuth and altitude angles, and their variations (delta), and the variation of the variation (delta delta) were recorded and calculated. The variation was calculated as the differences between adjacent elements in the time series. Variations are needed to obtain information such as acceleration, speed, instantaneous trajectory angle, instantaneous movement, tangential acceleration, curvature radius, and centripetal acceleration (Lopez-de-Ipina et al., 2016, 2018). Mean and standard deviation (std) were calculated for all these features and evaluated by PC to assess the control of fine movements (Figures 4, 5).
2. *Significant handwriting features and region of interest (ROI)* were evaluated by PC. In the putative motor circuit previously defined, correlations were tested for EtG-HW in cortical (CV and CT) and subcortical regions (CV). Finally, a hypothesis-driven ROI analysis of the motor Yeo functional networks in the cerebellum was tested (see “*Introduction*” section).

## Results

### Handwriting Analysis

**Figure 6:**
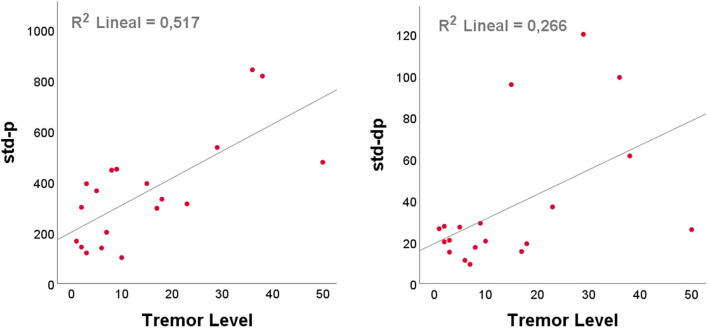
Scatterplots of handwriting features against tremor level with the best linear line fit, determined using ordinary least squares (OLS, SPSS): (left) standard deviation (std) of the pressure; (right) std of the pressure variation.

This subsection describes the handwriting features: spatial features, pressure, and their variation. Table 2 summarizes the results of partial correlation bilateral analysis, with brain volume correction, for the handwriting analysis and the tremor level. Significant features were the pressure standard deviation (std-p) and its variation and second variation (std-Δp, std-ΔΔp), which were directly related to the control of fine movements and plasticity, with a correlation of 0.719, 0.516 and 0.510, respectively. Figure 6 shows the details of: (i) std of pressure and (ii) std of pressure variation.

**Table 2:** Significant handwriting features assessed by Pearson’s partial correlation controlled for brain volume; significant values for *p*-value < 0.01 and *p*-value < 0.05 (bilateral).

		std-p	std-Δp	std-ΔΔp
Level	PC	0.717	0.563	0.552
	*p*-value	**0.001**	**0.015**	**0.018**

### Neuroimaging vs. Handwriting Biomarkers

This subsection describes the correlation between neuroimaging biomarkers and tremor level.

Table 3 summarizes the results of partial correlation bilateral analysis, with brain volume correction, for the structure of cortical areas with pressure and pressure variations. Significant areas with regard to the level appeared in the precentral area and mainly in the CT. Significant correlations appeared between the left precentral and std of the variation and control of pressure.

**Table 3:** Significant areas in Pearson’s partial correlation (PC) analysis with brain volume correction for the motor cortex (DSK 308 parcellation); significant values for *p*-value < 0.01 and *p*-value < 0.05 (bilateral): CV and CT.

		Level	std-p	std-Δp	std-ΔΔp
lh_precentral_part7	PC	−0.513	−0.355	−0.590	−0.524
	*p*-value	**0.030**	0.148	**0.010**	**0.026**
lh_precentral_part9	PC	−0.449	−0.447	−0.520	−0.501
	*p*-value	0.061	0.063	**0.027**	**0.034**
rh_precentral_part7	PC	−0.312	−0.413	−0.479	−0.452
	*p*-value	0.208	0.089	**0.044**	0.059
lh_ct_precentral_part7	PC	−0.518	−0.393	−0.198	−0.196
	*p*-value	**0.028**	0.107	0.431	0.436
rh_ct_precentral_part1	PC	−0.507	−0.432	−0.248	−0.297
	*p*-value	**0.032**	0.073	0.321	0.232
rh_ct_precentral_part4	PC	−0.468	−0.316	0.051	**−0.036**
	*p*-value	**0.050**	0.201	0.841	0.886
rh_ct_precentral_part6	PC	−0.477	−0.405	−0.101	−0.149
	*p*-value	**0.046**	0.095	0.690	0.556
rh_ct_precentral_part7	PC	−0.561	−0.487	−0.314	−0.317
	*p*-value	**0.015**	**0.040**	0.205	0.199

Table 4 summarizes the results of bilateral analysis of Pearson’s partial correlation for the structure of subcortical areas with pressure and pressure variations for the left hemisphere (lh) and the right hemisphere (rh). There were no significant subcortical areas with regard to the tremor level. However, significant correlations appeared with std of pressure and variations and basal ganglia (lh_Caudate, rh_Caudate, lh_Putamen, and rh_Putamen) as well as in rh_Thalamus.

**Table 4:** Pearson’s partial correlation (PC) analysis with brain volume correction for subcortical areas; significant values for *p*-value < 0.01 and *p*-value < 0.05 (bilateral).

		Level	std-p	std-Δp	std-ΔΔp
lh_Cerebellum_White_Matter	PC	−0.296	−0.407	−0.386	−0.334
	*p*-value	0.232	0.094	0.114	0.176
lh_Cerebellum_Cortex	PC	0.184	0.136	−0.136	−0.103
	*p*-value	0.466	0.591	0.590	0.685
lh_Thalamus	PC	−0.131	−0.156	−0.223	−0.266
	*p*-value	0.604	0.537	0.373	0.286
lh_Caudate	PC	**0.026**	0.168	0.480	0.482
	*p*-value	0.919	0.505	**0.044**	**0.043**
lh_Putamen	PC	0.116	−0.039	0.308	0.326
	*p*-value	0.645	0.878	0.214	0.187
lh_Pallidum	PC	0.138	**0.032**	0.285	0.276
	*p*-value	0.586	0.900	0.252	0.267
rh_Cerebellum_White_Matter	PC	−0.150	−0.350	−0.367	−0.305
	*p*-value	0.552	0.154	0.134	0.218
rh_Cerebellum_Cortex	PC	0.466	0.385	0.020	**0.051**
	*p*-value	0.051	0.114	0.938	0.840
rh_Thalamus	PC	−0.539	−0.690	−0.641	−0.573
	*p*-value	**0.021**	**0.002**	0.004	0.013
rh_Caudate	PC	0.114	0.259	0.517	0.440
	*p*-value	0.654	0.299	0.028	0.068
rh_Putamen	PC	0.092	0.220	0.439	0.351
	*p*-value	0.715	0.380	0.068	0.153
rh_Pallidum	PC	0.524	0.386	0.441	0.441
	*p*-value	**0.026**	0.114	0.067	0.067

Table 5 summarizes the results of Pearson’s partial correlation (bilateral) analysis for Yeo 7 and 17 functional motor networks in the cerebellum and the pressure and pressure variations. The most significant areas with regard to tremor severity appeared in lh_Somato_Motor_A for Yeo 17 functional networks. Significant correlations appeared in the cerebellum among std of pressure and variations and lh_Somato_Motor, rh_Somato_Motor, and lh_Somato_Motor_A.

**Table 5:** Pearson’s partial correlation analysis with brain volume correction for the Yeo networks in the cerebellum; significant values for *p*-value < 0.01 and *p*-value < 0.05 (bilateral).

		Level	std-p	std-Δp	std-ΔΔp
lh_Somato_Motor	PC	0.715	0.505	0.384	0.420
	*p*-value	**0.001**	**0.032**	0.115	0.082
rh_Somato_Motor	PC	0.527	0.562	0.338	0.435
	*p*-value	**0.025**	**0.015**	0.170	0.071
lh_Somato_Motor_A	PC	0.782	0.565	**0.508**	0.509
	*p*-value	**0.000**	**0.015**	**0.031**	**0.031**
lh_Somato_Motor_B	PC	**−0.033**	−0.086	−0.209	−0.093
	*p*-value	0.898	0.735	0.405	0.713
rh_Somato_Motor_A	PC	0.452	0.463	0.277	0.336
	*p*-value	0.059	0.053	0.266	0.172
rh_Somato_Motor_B	PC	0.173	0.189	0.175	0.290

**Figure 7:**
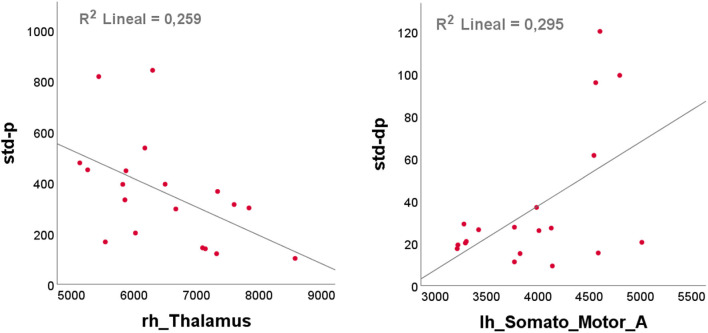
Scatterplots of handwriting features against neuroimaging biomarkers with the best linear line fit, determined using ordinary least squares (OLS, SPSS): (left) std of pressure and rh_Thalamus; (right) std of pressure variation and lh_Somato_Motor_A, a network in the cerebellum.

Figure 7 shows examples of significant Pearson correlations for handwriting features and neuroimaging biomarkers by scatterplots: (i) std of pressure and rh_Thalamus and (ii) std of pressure variation and lh_Somato_Motor_A network in the cerebellum.

## Discussion

In this study, a novel diagnostic approach for ET patients is presented based on two robust analysis strategies: (i) noninvasive analysis of Archimedes’ spiral for diagnosis (Lopez-de-Ipina et al., 2018) and (ii) structural neuroimaging analysis (Passamonti et al., 2012; Sharifi et al., 2014). The study is based on a unique dataset aimed at developing tools for early clinical diagnosis of ET; a range of patient age and tremor severities were included in order to best capture clinical variation. Analyses of advanced handwriting datasets are under-represented in the literature, and thus, our work represents a novel contribution to the field (Vessio, 2019).

Our hypothesis-driven analysis focused on three axes: (i) the motor circuit and functional networks in the cerebellum, exploring in detail the structural neuroimaging of the motor network circuitry: the motor cortex, the cerebellum, the thalamus, and the basal ganglia; (ii) fine motor skills, analysis trajectory variations, and pressure, and its fine variations in Archimedes’ spiral; and (iii) correlation between handwriting and neuroimaging biomarkers. On the other hand, due to the controversial role of the cerebellum in the pathophysiology of ET (Agarwal and Biagioni, 2020), this study also contributes to increasing evidence of the cerebellum as an important structure in patients with ET (Sharifi et al., 2014; Latorre et al., 2019).

The combination of both automated handwriting and neuroimaging analytics may represent a promising strategy in early detection, confirmation, and management of ET patients. Since there are no clear and specific biomarkers for ET, neuroimaging analysis may be harnessed to better distinguish ET from other tremor-related pathologies (Agarwal and Biagioni, 2020). Moreover, misdiagnosis among tremor syndromes is common and can impact on both clinical care and research (Jain et al., 2006). To date, no validated neurophysiological technique is available that has proven to have good classification performance above and beyond the clinical diagnosis made by a movement disorders expert. However, a handwriting sample can be used either as a noninvasive strategy to distinguish Parkinson’s disease (PD) and ET (Reich, 2020) or as a quantitative tool for the automated analysis of tremor severity (Elble et al., 1996). In fact, Archimedes’ spiral is one of the gold standard tests for ET analysis (Lopez-de-Ipina et al., 2018). Thus, the correlation and integration of biomarkers of both methodologies are essential. In this first validation, we explored the spiral as a support tool by correlating it with the gold standard clinical diagnosis and imaging alterations described by MRI. Indeed, these imaging analyses were consistent with the alterations described with the automated analysis of the spiral parameters. Interestingly, we showed for the first time in 19 patients with ET that there exists a significant correlation between the automatic analysis developed by the Archimedes’ spiral and the structural and functional changes described in the neuroimaging analysis according to the level of tremor severity.

Since the early detection of ET and the management of disease, not only in clinical but also in domestic environments, there has been a requirement for robust, noninvasive, easy-to-use approaches for the characterization of the disorder. These findings enable us to suggest Archimedes’ spiral as a noninvasive tool with high precision that could be used in supporting clinicians. Furthermore, the handwriting samples needed are easily available and can be acquired at low cost from tremor recordings for brief periods of time as well as provide a context-independent interpretation. Indeed, systems with automated analyses are being pursued in several studies with the purpose of obtaining an accurate and noninvasive diagnosis. On the other hand, neuroimaging analysis is one of the most robust assessments in neurological disorders and the automated analysis of handwriting is a robust and easy tool for use in clinical practice (Sharifi et al., 2014; Schuhmayer et al., 2017). In this context, a recent study also proposed the use of machine learning methods in combination with neuroimaging techniques for the early diagnosis of ET by measuring cortical thickness (Serrano et al., 2017). Accordingly, Sengul and colleagues have shown a correlation between brain microstructural changes of both white matter and gray matter with cognitive function in patients with ET, suggesting that other brain structures are related to the level of cognitive performance above and beyond the cerebello-thalamo-cortical pathway (Sengul et al., 2020).

Furthermore, it is speculated that the cerebellum plays an important role in the guidance and control of movement after receiving sensory information (Koziol and Budding, 2012). In this regard, a recent study has strongly supported the functional activity changes in the cerebello-thalamo-cortical network, which is mainly involved in the coordination of movements (Patel et al., 2014), in patients with ET during motor task performance. In fact, the authors suggest in the study that this network is also altered in rest conditions when tremor is absent (Nicoletti et al., 2020). Thus, it is fundamental to focus on the structural basis of the ET in order to functionally elucidate which regions are involved and altered in its pathophysiology in order to achieve a correct diagnosis and monitoring of further effects. In particular, our findings indicated that patients with ET had alterations in the cerebello-thalamo-cortical motor circuitry that resulted in loss of fine movement skills.

The results of the analysis support the hypothesis that the increase in severity of tremor (reflected in variations in pressure) correlates proportionally with the alteration in the cerebello-thalamo-cortical connectivity. In 2015, Buijink and colleagues demonstrated through a connectivity analysis a modulating effect of tremor variation on the cerebellum-dentato-thalamic connection and the intrinsic activity of the thalamus and cerebellum (lobe V). In a similar way to that described in previous studies (Raethjen et al., 2007), they found a decrease in cerebellar-cortical functional connectivity related to the performance of motor tasks. Interestingly, decreased functional coupling between the primary motor cortex and the posterior cerebellum was associated with an increase in tremor severity, and increased tremor intensity was associated with greater functional connectivity between the cerebellar lobes I–IV and the motor thalamus. That is, the altered output activity in the cerebellum would generate an inadequate thalamic activity, which would be able to interrupt the physiological connectivity with the motor cortex (Buijink et al., 2015). These data are consistent with the findings of the coherence study between EEG–EMG already mentioned (Raethjen et al., 2007), in which it was shown that cortical activity can be lost intermittently without changes in tremor suggesting that cortical involvement is not crucial in the genesis of tremor.

Overall, our study has the following key findings: (i) association between tremor level with pressure and its variations; (ii) correlation of tremor level to brain structure in several areas of the motor cortex (specifically, differences in cortical volume and cortical thickness); (iii) correlation among fine movement skills, pressure, and variations to subcortical areas, thalamus, ganglia, and cerebellum; and (iv) functional variation vs. structural changes analyzed by Yeo networks in the cerebellum and the correlation appearing in the somatomotor network with regard to tremor level, pressure, and variations.

Currently, the diagnosis of ET is clinically driven and there are no specific biomarkers available. In recent years, an important effort has been made to classify ET with respect to other diseases and physiological tremor (consensus statement on the classification of tremors, from the Task Force on Tremor of the International Parkinson and Movement Disorder Society; Bhatia et al., 2018). Cases of overt tremors are generally easy to diagnose, although there are serious problems in reaching diagnostic certainty when the intensity of the tremor is small. By refining the diagnostic threshold in the early stages of ET, we may better conduct studies with oligosymptomatic subjects in two key areas: (i) research on the segregation of candidate genes in familial tremor, allowing assessment of the phenotype in doubtful cases, which is very common in patients, even from the same family (Magrinelli et al., 2020); and (ii) research study designs in therapeutic trials for patient selection of participants and for their follow-up assessment.

The results of this study confirm the concordance of findings between the clinical examination and neuroimaging results, even in low amplitude tremors, thus positioning it as a tool to improve the performance of the neurological diagnosis of difficult cases (Filip et al., 2020). However, this study has some limitations. First, the wide range of tremor levels in the sample was selected at the expense of reduced sample size (*N* = 19). This sample size limits the power and full generalizability of the results (futility study), and hence, results must be validated in larger cohorts specifically designed for this purpose. However, the selection of hypothesis-driven biomarkers provides a robust experimental framework that adds value to the novel methodology and results.

## Conclusions

Essential tremor is a movement disorder of high prevalence that requires efficient clinical trials not only for early diagnosis but also for monitoring and appropriate management of treatments. In this sense, the integration of noninvasive and inexpensive tools (such as the automatic analysis of handwriting and drawing) along with robust biomarkers based on neuroimaging could become powerful support tools in future assessments. In addition, the correlation of both automated neuroimaging analysis with clinical findings allows us to consider the Archimedes’ spiral task as a valuable tool in the diagnosis and follow-up of ET. Ultimately, this work presents a novel approach based on automated analysis of Archimedes’ spiral and analysis of structural neuroimaging. The study involved patients with a wide range of tremor levels and explored fine variations in functionality over the motor circuit observed with structural neuroimaging. Results are promising and give a useful, easy-to-use, and robust support tool for the management of early tremor that can be easily integrated into current clinical assessments.

## Data Availability Statement

The datasets generated and analyzed during the current study are not publicly available due to ethics and privacy requirements but they could be available from the corresponding author KL-I on reasonable research request.

## Ethics Statement

The studies involving human participants were reviewed and approved by Ethics committee of the Donostia University Hospital. The patients/participants provided their written informed consent to participate in this study.

## Author Contributions

KL-I designed the study, designed and developed the features, software, dataset, performed the experiments, analyzed the results, prepared the figures, and wrote the manuscript. JS-C developed the features and software, analyzed the results, and wrote the manuscript. JS-M developed the software and dataset, performed the experiments, and analyzed the results. RR-G developed the features and software and analyzed the results. EF developed the software and dataset, analyzed the results, and prepared the figures. CR and AP analyzed the results and wrote the manuscript. MF-Z designed and developed the features, software, dataset, and analyzed the results. JM-M designed the study. AB designed the study, designed and developed the dataset, analyzed the results, and wrote the manuscript. JS designed the study and the features, analyzed the results, prepared the figures, and wrote the manuscript. All authors contributed to the article and approved the submitted version.

## Conflict of Interest

The authors declare that the research was conducted in the absence of any commercial or financial relationships that could be construed as a potential conflict of interest.
